# *In vivo* Protein Interference: Oral Administration of Recombinant Yeast-Mediated Partial Leptin Reduction for Obesity Control

**DOI:** 10.3389/fmicb.2022.923656

**Published:** 2022-06-14

**Authors:** Feng Yue, Lihong Du, Ruyu Wang, Baoquan Han, Xiaojun Zhang, Zhangzhang Yao, Wenqiang Zhang, Chang Cai, Zhiying Zhang, Kun Xu

**Affiliations:** College of Animal Science and Technology, Northwest A&F University, Shaanxi, China

**Keywords:** protein interference, protein knock-down, leptin resistance, partial leptin reduction, obesity control, oral administration, recombinant yeast

## Abstract

Obesity-related diseases are always the major health problems that concern the whole world. Serial studies have reported that obesity development is closely related to the out-of-control leptin encoded by the obesity gene (*ob*). The latest report declaimed “Less Is More,” a model explaining that partial leptin reduction triggers leptin sensitization and contributes to obesity control. Here, we came up with a novel concept, *in vivo* protein interference (iPRTi), an interesting protein knock-down strategy for *in vivo* partial leptin reduction. First, the specific immune response against leptin induced by the oral administration of *ob* recombinant yeast was confirmed. Subsequentally, leptin resistance was observed in diet-induced obese mice, and oral administration with *ob* recombinant yeast declined the circulating leptin and reduced significantly the body weight gain. To further investigate whether the iPRTi strategy is capable of obesity management, the diet-induced obese mice were administrated with *ob* recombinant yeast. All the indexes examined including the circulating leptin, triglyceride, and total cholesterol, as well as food intake and weight gain, demonstrated a positive effect of the iPRTi strategy on obesity control. In short, this study provides a novel strategy for the potential application of recombinant yeast for the therapy of obese individuals with leptin resistance.

## Graphical Abstract

Oral administration of *ob* recombinant yeast mediated partial leptin reduction for diet-induced obesity control. When the whole yeast is uptaken by phagocytic cells in the gut, for example, dendritic cells, it is supposed to be lysed within the cells. The recombinant DNA vaccine cassette can be released, expressed, and further presented to lymphocytes, thereby activating the immune response. 
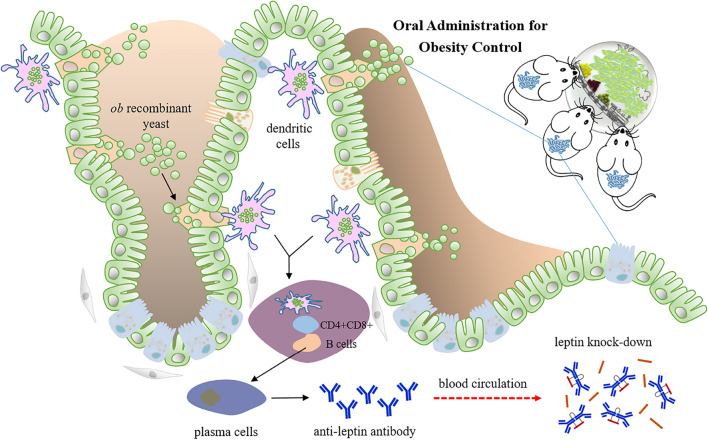

## Highlights

– Oral administration of *ob* recombinant yeast induces *in vivo* specific immune response.
– Oral administration of diet-inducing obese mice with *ob* recombinant yeast relieves leptin resistance and reduces body weight gain.
– Oral administration of diet-induced obese mice with *ob* recombinant yeast contributes to obesity control.

## Introduction

Obesity mainly characterized by excess body fat is one of the prominent risks causing chronic diseases, including type 2 diabetes, atherosclerosis, cardiovascular disease, cancer, and other related diseases It has become a major health problem covering the world. Although numerous efforts have been made for obesity control, including lifestyle change, surgical treatment, medical therapy, and so on, the weight loss effects often fail to meet the demand of obese individuals. What’s more, pharmacological leptin therapy for the treatment of diet-induced obesity has been reported to be ineffective, which is the famed “leptin resistance” phenomenon (Zhao et al., 2020a).

Leptin, encoded by the obese gene (*ob*), is known as an adipocyte hormone that controls energy expenditure, and acts as an afferent negative feedback signal to the central nervous system, maintaining homeostatic control of adipose tissue mass and regulating appetite and metabolism (Zhang et al., 1994). Leptin is secreted primarily by white adipose tissue and its secretion is proportional to adipocytes (Maffei et al., 1995). Many studies have revealed that increased leptin secretion is a significant predictor of altered blood lipid profiles and glucose–insulin homeostasis (Frederich et al., 1995; Laforest et al., 2015). In the context of conventional obesity, obese individuals do not lack leptin; rather, they display higher levels of circulating leptin. However, the oversecreted leptin will not contribute to energy expenditure, diet control, and weight loss, which is known as leptin resistance and is caused by impaired leptin signaling in the brain (Zelissen et al., 2005; Knight et al., 2010; Flier and Maratos-Flier, 2017).

Therefore, “Less Is More,” partial leptin reduction has been raised recently as an insulin sensitization and weight loss strategy (Zhao et al., 2020a). The latest studies have demonstrated that insulin sensitization and weight loss could be achieved by reducing circulating leptin through an immunological approach in an obesogenic environment (Zhao et al., 2019, 2020b; Hankir and Seyfried, 2020). Neutralizing antibody injection triggers an immune response to endogenous oversecreted leptin, and maintaining lower circulating leptin is highly beneficial to obesity control in diet-induced obese mice (Zhao et al., 2020b).

Inspired by the idea to trigger the immune response to endogenous oversecreted leptin for obesity control, we proposed in the current study to develop an oral yeast vaccine for leptin knock-down based on our previous studies and came up with a novel concept *in vivo* protein interference (iPRTi).

## Materials and Methods

### Strains and Plasmids

*Saccharomyces cerevisiae* JMY31 strain (*MAT*α, *ade2-1; ura3-1; his3-11; trp1-1; leu2-3,112; can1-100*) and *Escherichia coli* JM109 and Rosetta (DE3) strains, as well as the JMB84 plasmid, all came from the lab storage as we previously used (Han et al., 2019; Zakria et al., 2019). JMB84 is a yeast-bacteria shuttle vector harboring replication origins and selecting marker genes intent for both yeast and *Escherichia coli* transformation. It was used as the backbone and the negative control in the current study. The JMY31 strain for the construction of recombinant yeast vaccines was grown and kept in a YPD medium. The JM109 strain for the construction of recombinant plasmids and The Rosetta (DE3) strain for leptin prokaryotic expression were grown and kept in an LB medium.

### Construction of Vectors

**FIGURE 1:**
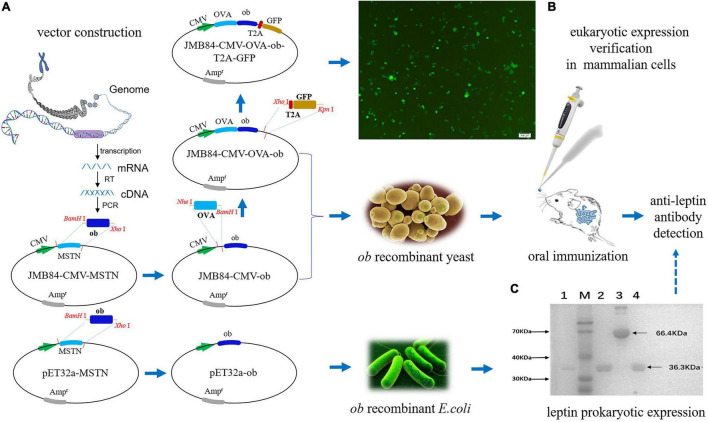
Schematic diagram of the experiment design and vector verification. **(A)** Flow chart for the construction of the plasmid vectors. **(B)** Fluorescence observation of the 293T cells transfected with the JMB84-CMV-OVA-ob-T2A-GFP vector. The GFP expression suggested the recombinant CMV-OVA-*ob* cassette, as a DNA vaccine, could be expressed successfully in mammalian cells. **(C)** SDS-PAGE electrophoresis result of the purified leptin protein. Line 1, 2, and 4: purified leptin proteins expressed by different *E. coli* Rosetta (DE3) transformants. Line 3, the bull serum albumin (BSA) control. M: Blue Plus II protein marker.

To establish the iPRTi and verifying system, a series of plasmid vectors as shown in Figure 1A was constructed by recombinant DNA technology using JM109 competent cells. The coding sequence (CDS) of the mouse *ob* gene (NM_008493.3) was first cloned from the adipose tissue cDNA by RT-PCR. The yeast shuttle vector JMB84-CMV-ob and the prokaryotic expression vector pET32a-ob were generated, respectively, by replacing the *MSTN* CDS of the JMB84-CMV-MSTN (Zakria et al., 2019) and pET32a-MSTN (Zhang et al., 2011) vectors with the *ob* CDS. Based on the JMB84-CMV-ob vector, the *OVA* CDS and the *T2A-GFP* open reading frame were cloned up- and downstream of the *ob* CDS successively to generate the JMB84-CMV-OVA-ob and JMB84-CMV-OVA-ob-T2A-GFP vectors. Similar to what we previously did (Han et al., 2019), the ovalbumin (OVA) was introduced to enhance the immunogenicity, and the green fluorescent protein (GFP) was used to verify the expression of the *ob* DNA vaccine cassette in mammalian cells.

### Verification of the *ob* Recombinant Cassette in Mammalian Cells

Mammalian 293T cells were maintained in Dulbecco’s modified Eagle’s medium (DMEM; GIBCO) supplemented with 10% fetal bovine serum (FBS; BI), 100 U/ml penicillin, and 100 mg/ml streptomycin in a 37^°^C humidified atmosphere with 5% CO_2_ incubation. The cells were seeded into 12-well plates and were transfected with the JMB84-CMV-OVA-ob-T2A-GFP plasmid DNA by Lipofectamine 2000 (Invitrogen) according to the manufacturer’s protocol. After 48 h of the transfection, the cells were observed to confirm the expression of the CMV-OVA-ob-T2A-GFP recombinant cassette and were photographed with a fluorescence microscope.

### Preparation of the Recombinant Whole Yeast Vaccines

Yeast JMY31 cells were transformed using the LiAc method (Gietz, 2014), respectively, with the shuttle vectors JMB84-CMV-ob and JMB84-CMV-OVA-ob, as well as the control empty vector JMB84. Different transformant clones were selected using plates with SD/-Ura medium (Clontech) incubated at 30^°^C for 3 days and were further expanded in liquid SD/-Ura medium with shaking for another 3 days. The yeast transformant cells were harvested, resuspended in PBS with the density of 2 × 10^8^ cfu/ml and heat-inactivated at 56^°^C for 1 h (Kiflmariam et al., 2013). The resuspended and inactivated yeast transformant cells were then aliquoted and stored at −80^°^C until the oral administration.

### Prokaryotic Expression of Leptin

*Escherichia coli* Rosetta (DE3) transformants with the recombinant plasmid pET32a-ob were selected using LB plates with ampicillin supplemented. The transformant cells were seeded and expanded in liquid LB/+Amp medium at 37°C with shaking. When the optical density at 600 nm (OD_600_) reached up to 0.6, the expression was induced with 0.5 Mm IPTG supplemented for 4 h. Then, the bacterial cells were harvested, resuspended in PBS, and subjected to sonication. The protein purification of recombinant leptin was conducted using the AKTA protein chromatography system with Ni-NTA Kit (BaiHui, China) according to the manufacturer’s protocol. The purified recombinant leptin was stored at −80°C for the subsequent detection of serumal anti-leptin antibodies.

### Mice and Oral Immunization

A total of 96 5-week-old female Kunming mice were purchased from the Breeding and Research Centre of Xi’an Jiaotong University, China. All mice were housed and handled according to the guidelines for the Welfare and Moral Inspection of Experimental Animals in China (No. 2 Order of State Science and Technology Commission of the PRC) and were approved by the Animal Ethics Committee of Northwest A&F University (approval number NWAFU-DK2020046). Generally, the mice were raised with a 12-h light/12-h dark cycle and in a temperature-controlled environment (22 ± 2^°^C) and had *ad libitum* access to food and water.

**FIGURE 2:**
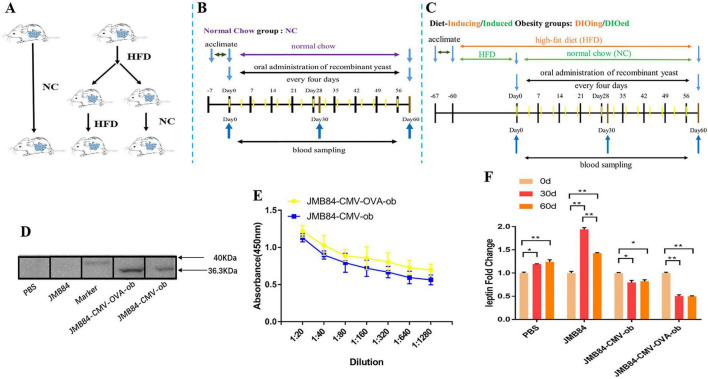
Oral immunization groups and timelines, preliminary immune response induced by oral administration of *ob* recombinant yeast in NC mice. **(A)** Oral immunization groups. **(B)** The feeding, oral immunization, and blood sampling timeline for the NC group. **(C)** The feeding, immunization, and sampling timeline for the DIOing and DIOed groups. **(D)** WB assay result with purified leptin recombinant protein as the antigen and different antiserums (Day60) from different treatments as the primary antibodies. **(E)** ELISA result for anti-leptin antibody titering in the antiserums (Day60) from the oral immunized mice by *ob* recombinant yeast. **(F)** The changes in circulating leptin were detected by ELISA in the antiserums (Day0, Day30, and Day60) from different treatments. The * sign indicates statistical significance at a *P* value of < 0.05; ** represents *P* < 0.01.

For the oral immunization experiment (as shown in Figures 2A–C), all the mice were acclimated to the new environment for 1 week after arrival and were randomly divided into the normal chow (NC) group and the high-fat diet (HFD) groups. The HFD groups included the diet-inducing obesity (DIOing) group maintained with continuous D12492 high-fat feeding and the diet-induced obesity (DIOed) group with NC after high-fat feeding induction (Figure 2C). Each group was further divided into four treatments (as shown in Table 1) with oral administration of different recombinant yeast and control PBS solution. The experiment mice for each treatment were housed in two cages and were orally administrated with recombinant yeast 100μl once per mouse every 4 days for a total of 60 days. Blood samples were collected by tail vein sampling before, during, and after the immunization (Day0, Day30, and Day60). The samples of the same time from different individuals of each treatment were mixed for antiserum preparation.

**TABLE 1:** Oral administration treatments for each experiment group.

Treatment	Oral administration	Density	Dosage	Function
PBS	–	–	100 μl	Blank control
JMY31 (JMB84)	Whole yeast	2 × 10^8^ cfu/ml	100 μl	Carrier control
JMY31 (JMB84-CMV-ob)	Whole yeast (*ob*)	2 × 10^8^ cfu/ml	100 μl	Vaccine group
JMY31 (JMB84-CMV-OVA-ob)	Whole yeast (*OVA-ob*)	2 × 10^8^ cfu/ml	100 μl	Vaccine group

### Body Weight and Food Intake Measurement

In order to measure the changes in the body weight and food intake of the mice in the HFD groups, the mice of different treatments were acclimated for at least 1 week to reduce the anxiety effects, and the initial body weight before oral immunization was measured. Then, the body weight and food intake during the experiment were measured every week. The data for food intake was represented as a daily average. The body weights of the mice from different treatments were measured every week, and the average body weight gain was calculated at the end of oral immunization.

### Western Blotting Assay

The western blotting (WB) assay was conducted for the preliminary qualitative validation of anti-leptin antibodies in the antiserum from immunized mice. The purified recombinant leptin protein prepared above by prokaryotic expression was used as an antigen for the WB detection. The antiserums (Day60) from immunized mice of different treatments served as the primary antibodies at a 1:50 dilution. A goat-anti-mouse secondary antibody labeled with HRP was used at the dilution 1:2,000. The results were visualized with chemiluminescence by the ChemiDoc XRS imaging system (Bio-Rad, United States).

### Enzyme-Linked Immunosorbent Assay

The Enzyme-linked immunosorbent assay (ELISA) for tittering the anti-leptin antibodies in the antiserums was performed as we previously did (Zakria et al., 2019). 96-well EIA/RIA plates were coated overnight at 4^°^C with the purified recombinant leptin protein (100 μl, 5 ng/μl) and blocked for 2 h at 37^°^C with 5% non-fat milk. After washed with PBS 3 times, serial diluted antiserums (Day60, 1:20; 1:40; 1:80; 1:160; 1:320; 1:640; 1:1280) were added and incubated for overnight at 4^°^C. Subsequently, the plates were washed with PBS and incubated for 2 h with a goat anti-mouse HRP-conjugated antibody (Beyotime, China). The immunoreactions were developed with TMB substrates (Tiangen, China) and stopped by the addition of 2 M sulfuric acid before the plate was read at 450 nm. The circulating leptin in the antiserums (Day0, Day30, and Day60) was also detected using a commercial ELISA kit according to the user manual and the results were calculated by standard curve (Solarbio, China). Each antiserum mixture was assayed with at least three technical repeats.

### Total Cholesterol and Triglyceride Tests

Total cholesterol and triglyceride test kits were purchased from Nanjing Jiancheng Bioengineering Institute^1^ and the tests were performed according to the proposal. The reagent was mixed with the antiserum and incubated at 37^°^C for 10 min before the plates were read at 510 nm by a micro plate spectrophotometer. Each antiserum mixture was assayed with at least three technical repeats.

### Liver Histology

Liver tissues from different immunized mice of the DIOing group were excised and fixed overnight in 4% paraformaldehyde and were thereafter switched to 50% ethanol for long-time storage. Then the liver tissues were paraffin embedded, sectioned, and sliced at 8 mm per slide. The slices were stained with hematoxylin-eosin (HE) and then visualized under a microscope (OLYMPUS CKX53).

### Statistical Analysis

Statistical analysis was evaluated with ANOVA by IBM SPSS Statistics 21. Data were expressed as mean ± standard error of mean (SEM). *p < 0.05 and ^**^p < 0.01 were regarded as statistically significant, and ns as no significance.

## Results

### Experiment Design and Vector Verification

In general, to establish the iPRTi and verifying system, the yeast shuttle vector JMB84-CMV-ob and the prokaryotic expression vector pET32a-ob were first constructed based on previous vectors (Figure 1A). The vector JMB84-CMV-OVA-ob was next constructed with OVA introduced to enhance the immunogenicity as we previously did (Han et al., 2019). JMB84-CMV-OVA-ob-T2A-GFP was further constructed with GFP introduced to verify the expression of the *ob* DNA vaccine cassette in mammalian cells. As OVA-ob was expressed as a fusion and JMB84-CMV-OVA-ob was constructed based on JMB84-CMV-ob with the same expression pattern, hence mammalian-expression observed from JMB84-CMV-OVA-ob-T2A-GFP by green fluorescence could prove the expression of both the CMV-OVA-ob and CMV-ob cassettes.

The whole experiment design was divided into three parts, respectively, mammalian expression verification, oral yeast immunization, and antigen prokaryotic expression. Firstly, 293T cells were transfected with the JMB84-CMV-OVA-ob-T2A-GFP plasmid DNA to confirm the functional expression of the CMV-OVA-ob-T2A-GFP cassette. The green fluorescence (Figure 1B) suggested the recombinant CMV-OVA-ob and CMV-ob cassettes, as DNA vaccines, which were the core components in this study, could be expressed successfully in mammalian cells. Secondly, yeast JMY31 cells were transformed with the shuttle vectors JMB84-CMV-ob and JMB84-CMV-OVA-ob, as well as the control empty vector JMB84. The corresponding transformants were selected, identified, and expanded for subsequent oral immunization. Meanwhile, *E. coli* Rosetta (DE3) transformants harboring pET32a-ob were used for the prokaryotic expression of leptin protein. As displayed in Figure 1C, the recombinant leptin was successfully expressed and purified, which would serve as the antigen in the WB and ELISA assays for the detection of serumal anti-leptin antibodies. As for the immunization, when the oral administrated whole yeast is uptaken by phagocytic cells in the gut, for example, dendritic cells (DCs), it is supposed to be lysed within the cells. The recombinant CMV-OVA-ob or CMV-ob cassettes can be released, expressed, and further presented to lymphocytes, thereby activating the immune response. Here, the recombinant CMV-OVA-ob or CMV-ob cassettes function as DNA vaccines and the yeast cell acts as the carrier.

### Oral Administration of *ob* Recombinant Yeast Induces a Specific Immune Response

To conduct the oral immunization experiment, all the mice were randomly divided into the NC group and HFD groups (Figure 2A), and the HFD groups were further termed as the DIOing and DIOed groups characterized by different high-fat feeding patterns. The feeding, oral immunization, and blood sampling timelines for the three groups are shown in Figures 2B,C.

Preliminary, to confirm specific immune response could be induced by oral *ob* recombinant yeast, the NC group mice were first orally administrated with different recombinant yeast. WB assay was first conducted for the qualitative validation of anti-leptin antibodies in the antiserums after the oral immunization. The result clearly demonstrated specific anti-leptin antibodies could be induced by the oral administration of both JMY31 (CMV-ob) and JMY31 (CMV-OVA-ob) yeast transformants (Figure 2D). ELISA assay was subsequently conducted for anti-leptin antibody titering, and the result (Figure 2E) shows that the anti-leptin antibody titer was slightly higher for the JMY31 (CMV-OVA-ob) treatment, suggesting introducing the *OVA* gene into a DNA vaccine could contribute to improving the specific immune effect. The circulating leptin detection result (Figure 2F) further demonstrated partial leptin reduction, which we would like to term as leptin knock-down or iPRTi, did can be achieved by the oral *ob* recombinant yeast. The knock-down effect was obviously more significant for the JMY31 (CMV-OVA-ob) treatment, which reached an exciting 50% after the 30- and 60-day intermittent immunizations. Besides, we noticed that the circulating leptin was increased for the control PBS and JMY31 (JMB84) treatments, which was supposed due to the potential overnutrition of “NC die” that we used.

### Oral Administration of *ob* Recombinant Yeast Relieves Leptin Resistance in DIOing Mice

To investigate whether the oral recombinant yeast meditated iPRTi strategy could contribute to leptin resistance remission, the mice of the DIOing group were administrated continuously with HFD feed before and during the oral immunization (Figure 2C). Leptin resistance could be induced routinely by a 60-day HFD induction, and continuous HFD feeding during the oral immunization was supposed to maintain the induction.

**FIGURE 3:**
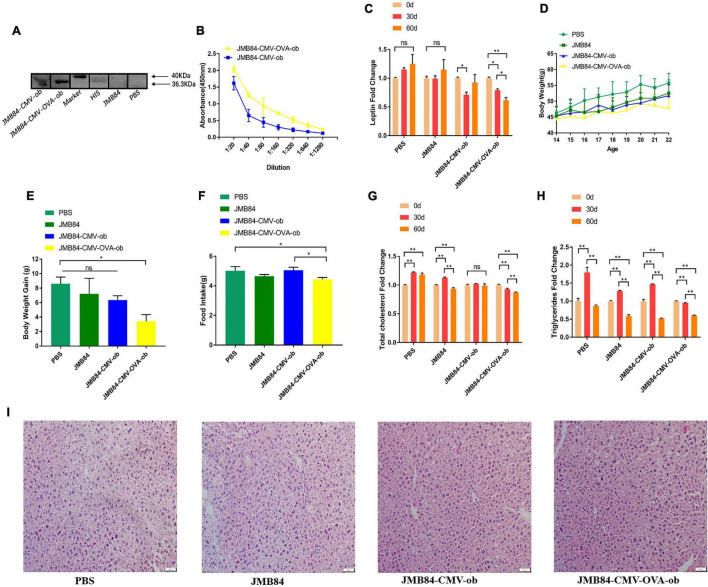
Oral administration of *ob* recombinant yeast relieves leptin resistance in DIOing mice. **(A)** WB assay result for the qualitative detection of anti-leptin antibodies in different antiserums (Day60) from different treatments. **(B)** ELISA result for anti-leptin antibody titering in the antiserums (Day60) from the oral immunized mice by *ob* recombinant yeast. **(C)** The changes of circulating leptin detected by ELISA in the antiserums (Day0, Day30, and Day60) from different treatments. **(D)** The weekly average body weight of the mice from different treatments. The age was indicated as weeks, and the mice were orally immunized at 14-week-old. **(E)** The comparison of the average body weight gain of the mice from different treatments at the end of oral immunization (Day60). **(F)** The comparison of the average daily feed intake of the mice from different treatments during the oral immunization. **(G,H)** The changes in total cholesterol and triglyceride were detected in the antiserums (Day0, Day30, and Day60) from different treatments. **(I)** Histological analysis of mice liver from different treatments. The “ns” indicates statistical significance at a *P* value of > 0.05; * represents *P* < 0.05; ** represents P < 0.01.

The WB and ELISA assay results both demonstrated again that specific immune response could be induced by oral *ob* recombinant yeast and JMY31 (CMV-OVA-ob) performed much better (Figures 3A,B). Further detection of the circulating leptin (Figure 3C) demonstrated firstly no significant increase during the oral immunization for the control treatments, indicating probably a highest level was reached after the 60-day HFD induction and leptin resistance were successfully induced, and secondly oral administration of the JMY31 (CMV-OVA-ob) recombinant yeast could reduce the circulating leptin effectively to about 75% in the context of continuous HFD induction.

In addition, the mice from the JMY31 (CMV-OVA-ob) treatment grew much more slowly than those from the control treatments (Figure 3D), and the corresponding body weight gain was reasonably the lowest (Figure 3E). The average daily feed intake of the mice from the JMY31 (CMV-OVA-ob) treatment was decreased compared to other treatments (Figure 3F), which was supposedly due to the leptin sensitization successfully triggered as expected by its partial reduction. Correspondingly, the blood total cholesterol and triglyceride were both downregulated significantly after the 60-day oral administration of the recombinant yeast JMY31 (CMV-OVA-ob; Figures 3G,H). HFD feeding usually affects tissue in a negative manner, especially liver, and we observed a marked reduction in hepatic steatosis after the 60-day oral administration of the *ob* recombinant yeast (Figure 3I).

### Oral Administration of *ob* Recombinant Yeast Contributes to Diet-Induced Obesity Control

In view that diet-induced obese individuals are not always fed with high-fat food and a normal diet is usually preferred when one is planning to lose weight, to further investigate whether the iPRTi strategy is capable practically for obesity management, we also conducted the DIOed group experiment with the diet-induced mice administrated with NC and *ob* recombinant yeast (Figure 2C).

**FIGURE 4:**
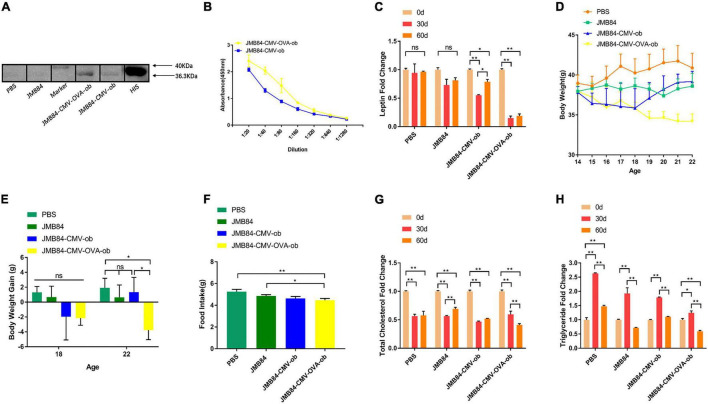
Oral administration of *ob* recombinant yeast contributes to obesity control of DIOed mice. **(A)** WB assay result for the qualitative detection of anti-leptin antibodies in different antiserums (Day60) from different treatments. **(B)** ELISA result for anti-leptin antibody titering in the antiserums (Day60) from the oral immunized mice by *ob* recombinant yeast. **(C)** The changes in circulating leptin were detected by ELISA in the antiserums (Day0, Day30, and Day60) from different treatments. **(D)** The weekly average body weight of the mice from different treatments. The age was indicated as weeks, and the mice were orally immunized at 14-week-old. **(E)** The comparison of the average body weight gain of the mice from different treatments at the middle (Day28, 18-week-old) and the end (Day56, 22-week-old) of oral immunization. **(F)** The comparison of the average daily feed intake of the mice from different treatments during the oral immunization. **(G,H)** The changes of total cholesterol and triglyceride were detected in the antiserums (Day0, Day30, and Day60) from different treatments. The “ns” indicates statistical significance at a *P* value of > 0.05; * represents *P* < 0.05; ** represents *P* < 0.01.

Same as above, the specific anti-leptin antibodies induced by oral *ob* recombinant yeast were first qualitatively and quantitatively confirmed by WB and ELISA, respectively (Figures 4A,B). Encouragingly, a dramatic reduction of the circulating leptin, which was knocked-down by more than 75%, was again detected after both 30- and 60-day oral administration of the recombinant yeast JMY31 (CMV-OVA-ob). The body weight of the mice from the JMY31 (CMV-OVA-ob) treatment was decreased 4 weeks (at the 18-week-old) after the oral immunization (Figures 4D,E), and the average daily feed intake was also decreased significantly (Figure 4F). Surprisingly, the blood total cholesterol was downregulated dramatically in all the treatments (Figure 4G), which was supposedly due to the diet changing to NC after HFD induction. We also observed an interesting rise-fall pattern for the blood triglyceride (Figure 4H), indicating a long-term 60-day oral immunization is necessary for diet-induced obesity management.

## Discussion

Yeast exhibits many particulate features of immunostimulatory complexes, and its cell-wall components, especially β-1,3-D-glucan and mannan, possess naturally immunologic adjuvant potential (Lee et al., 2021). The whole recombinant yeast vaccine, which was first put forward by Stubbs et al. (2001), can activate DCs to elicit protective cell-mediated immunity. DCs are the most powerful antigen-presenting cells in the body, and a little amount of DCs can stimulate a robust immune response (Palucka and Banchereau, 2012; Nava et al., 2021). In addition to the recombinant antigen, it has been reported that yeast does not induce any antibody response against its native proteins (Hudson et al., 2016). A lot of attention have been paid to the whole recombinant yeast vaccine for the potential application in the immunological therapy of cancer diseases (Miquel-Clopes et al., 2019).

On this basis, our laboratory has been devoted to the development of the oral yeast vaccine in the last decades. The whole recombinant yeast vaccine by oral administration was supposed to target M cells and DCs in the intestinal mucosal immune system. Targeted delivery to intestinal DCs of *in situ* expressed protein antigens (Zhang et al., 2011, 2012; Liu et al., 2016) and recombinant DNA vaccines (Kiflmariam et al., 2013; Yan et al., 2015; Han et al., 2019), as well as functional shRNA expression cassettes (Zhang et al., 2014; Xu et al., 2016; Zakria et al., 2019), were all achieved by oral administration of recombinant yeast in our previous studies.

Oral vaccine can induce systemically as well as mucosal immunity and is more attractive than an injectable vaccine for the pain-free vaccination route. However, gastrointestinal barriers and antigen degradation are the major concerns when developing oral vaccines (Gonzalez-Cruz and Gill, 2021). Yeast cell-wall, in addition to its natural adjuvant potential, can also play as a perfect immune protectant against gastrointestinal digestion. What’s more, our previous studies have demonstrated that the oral recombinant yeast vaccine is capable to trigger the immune response to not only exogenous antigens (Kiflmariam et al., 2013; Han et al., 2019), but also to endogenous factors such as myostatin (MSTN; Zhang et al., 2011; Zakria et al., 2019). Hence, the novel iPRTi strategy by oral administration of recombinant yeast vaccine was proposed in the current study and was further proved to be effective for *in vivo* partial leptin reduction.

Another major concern for oral vaccines is their relatively low efficiency. OVA was introduced in our previous studies to enhance the immunogenicity of oral recombinant yeast mediated both *in situ* protein vaccine (Zhang et al., 2011) and recombinant DNA vaccine (Han et al., 2019). The results in the present study further demonstrated clearly that the introduction of the *OVA* gene into a DNA vaccine cassette can improve its immune effect significantly. It has been also reported functional shRNA and miRNA could be delivered to the intestinal immune system by oral administration of recombinant yeast (Zhang et al., 2014, 2020, 2021). Another study we did previously further suggested that oral recombinant yeast vaccine could be enhanced by coupling with an shRNA for modulating DC-mediated immune response (Zakria et al., 2019). On the other hand, yeast cell surface β-glucan modification by genetic engineering, as well as antigen or/and ligand surface display, may be alternative strategies for improving the efficiency of oral recombinant yeast vaccines (Lei et al., 2020; Lee et al., 2021; Trentelman et al., 2021).

To guarantee the immune effect in this study, a 60-day long-term intermittent oral immunization was applied for all three experimental groups. First, in the NC group mice, the leptin knock-down effect was almost the same on Day30 and Day60 after the immunization initiation (Figure 2F). Secondly, in the context of continuous HFD induction (DIOing group), the whole 60-day immunization was demonstrated to be necessary for efficient leptin knock-down (Figure 3C), and the body weight growth was slowed down but not lost (Figure 3D). Finally, in the DIOed group mice administrated with NC diet after HFD induction, dramatical leptin knock-down (Figure 4C), as well as body the weight loss (Figure 4D), were observed in the JMY31 (JMB84-CMV-OVA-ob) treatment at both Day30 and Day60. Taken together, we conclude that oral administration of *ob* recombinant yeast can contribute to diet-induced obesity control, and synergetic dietary control is still necessary.

In conclusion, we present a novel iPRTi strategy for *in vivo* protein knockdown by oral administration of recombinant yeast, which was demonstrated to be effective for partial leptin reduction and diet-induced obesity control.

## Data Availability Statement

The original contributions presented in the study are included in the article/Supplementary Material, further inquiries can be directed to the corresponding author/s.

## Ethics Statement

The animal study was reviewed and approved by Animal Ethics Committee of Northwest A&F University (approval number NWAFU-DK2020046).

## Author Contributions

KX, ZZ, and FY contributed to the study design and data interpretation. FY was the principal investigator and wrote the draft. LD and KX contributed to the manuscript revising and figure drawing. All other authors contributed to partial experiment conduction and data analysis. All authors read and approved the final manuscript.

## Conflict of Interest

The authors declare that the research was conducted in the absence of any commercial or financial relationships that could be construed as a potential conflict of interest.

## Publisher’s Note

All claims expressed in this article are solely those of the authors and do not necessarily represent those of their affiliated organizations, or those of the publisher, the editors and the reviewers. Any product that may be evaluated in this article, or claim that may be made by its manufacturer, is not guaranteed or endorsed by the publisher.

## Footnotes

1
www.njjcbio.com
