# Supplementary material for: In vivo Protein Interference: Oral Administration of Recombinant Yeast-Mediated Partial Leptin Reduction for Obesity Control
Source: Front Microbiol. 2022 Jun 14;13:923656. doi: 10.3389/fmicb.2022.923656 (PMC9237534; doi:10.3389/fmicb.2022.923656)

WB original images of the NC group

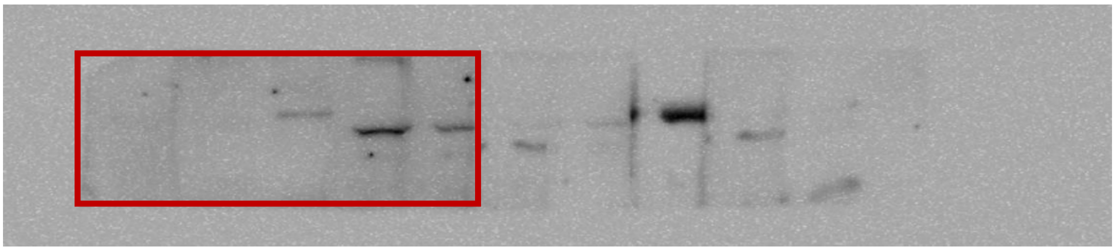

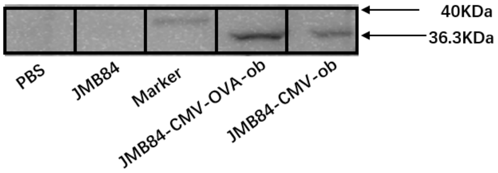

WB original images of the DIOing group

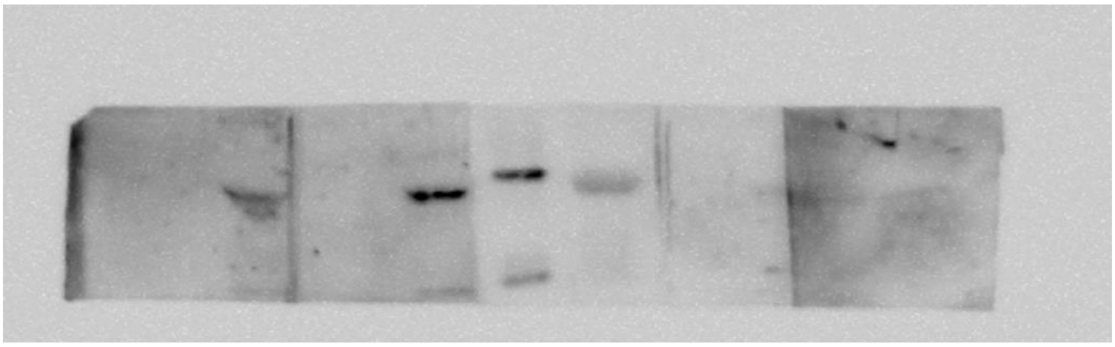

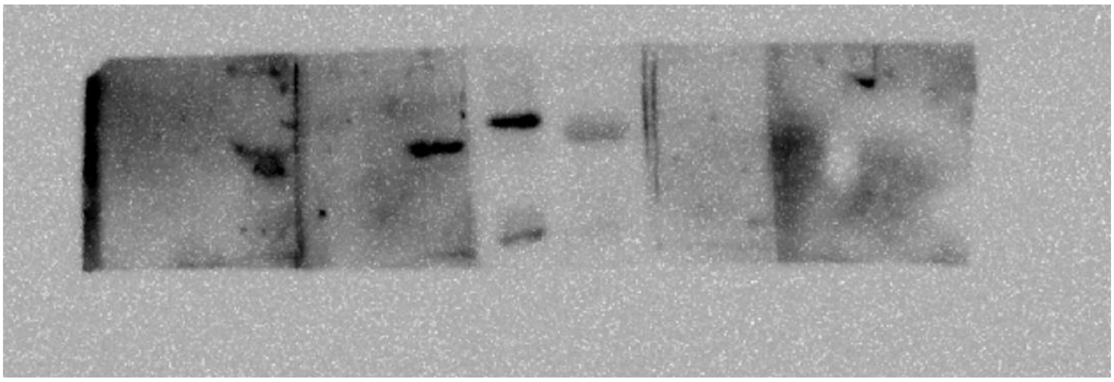

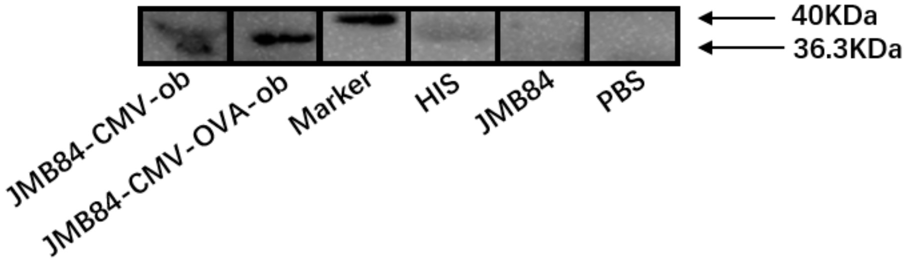

WB original images of the DIOed group

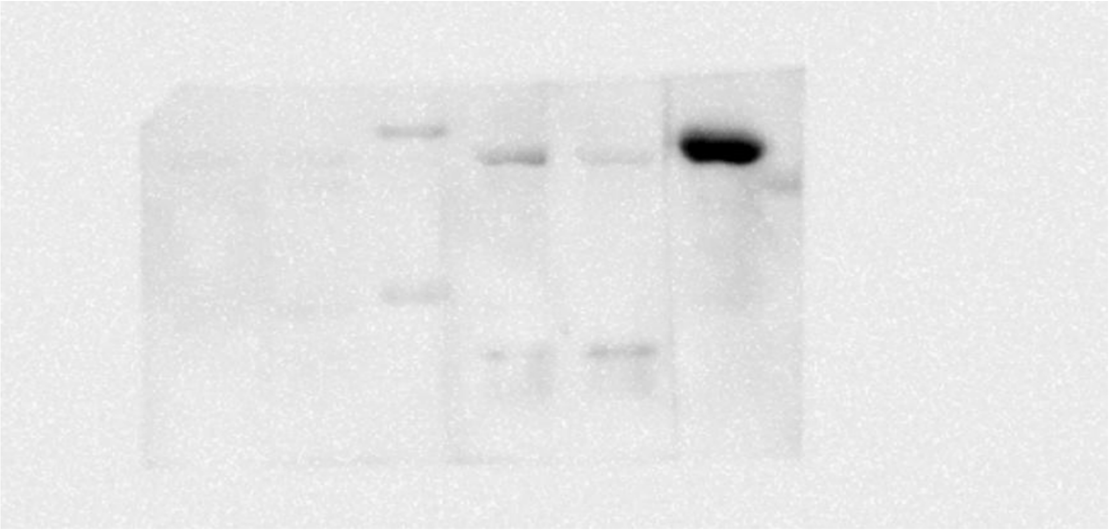

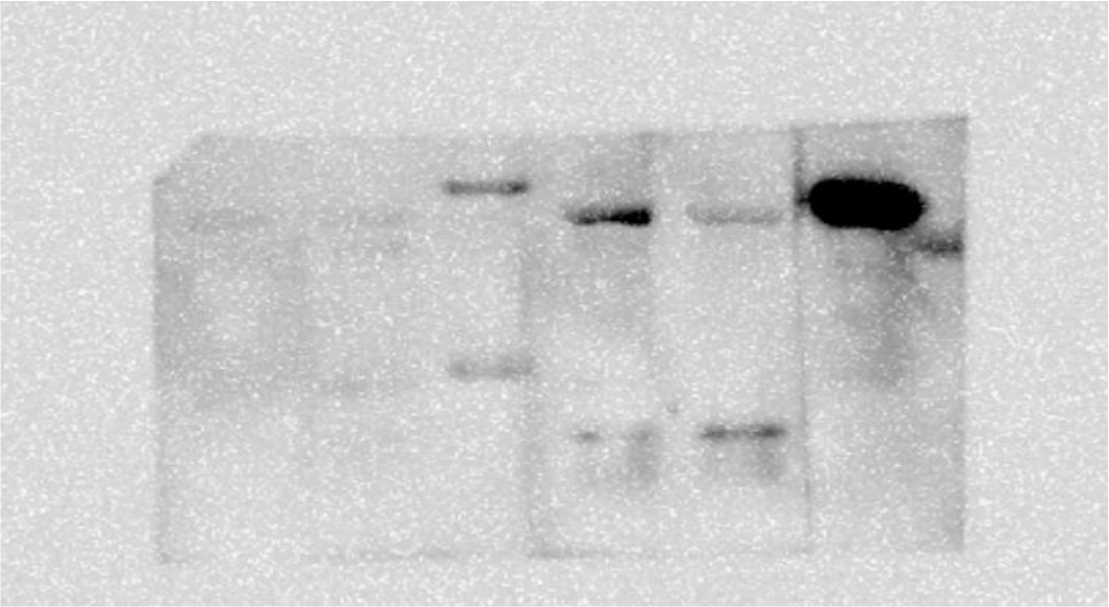

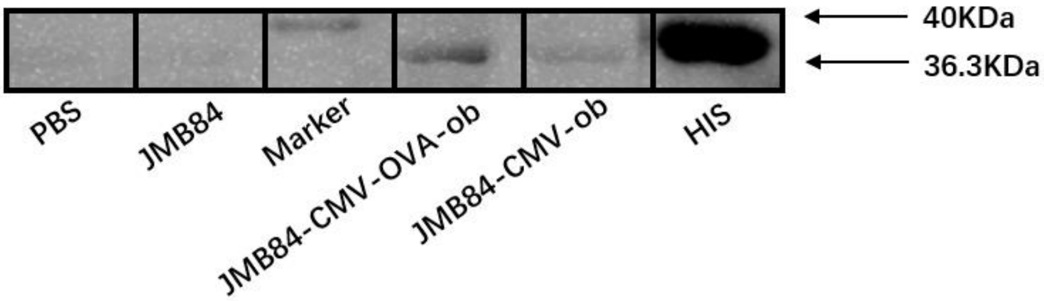

Supplement: Supplementary file 1 [file Data_Sheet_1.PDF]
